# Land use alters diazotroph community structure by regulating bacterivores in Mollisols in Northeast China

**DOI:** 10.3389/fmicb.2022.941170

**Published:** 2022-07-15

**Authors:** Zhiming Zhang, Xiaozeng Han, Fengjuan Pan, Hang Liu, Jun Yan, Wenxiu Zou, Neil B. McLaughlin, Xiangxiang Hao

**Affiliations:** ^1^Key Laboratory of Mollisols Agroecology, Northeast Institute of Geography and Agroecology, Chinese Academy of Sciences, Harbin, China; ^2^University of Chinese Academy of Sciences, Beijing, China; ^3^Key Laboratory of Soil Resource Sustainable Utilization for Jilin Province Commodity Grain Bases, College of Resource and Environmental Science, Jilin Agricultural University, Changchun, China; ^4^Ottawa Research and Development Centre, Agriculture and Agri-Food Canada, Ottawa, ON, Canada

**Keywords:** soil food webs, bacterivores, diazotroph community structure, ecological correlation, land use

## Abstract

Changes in land use can generate environmental pressures that influence soil biodiversity, and numerous studies have examined the influences of land use on the soil microbial communities. However, little is known about the effects of land use on ecological interactions of soil microbes and their predators. Diazotrophs are key soil microbes that play important functional roles in fixing atmospheric nitrogen. In this study, we investigated the co-association of diazotroph community members and patterns of diazotroph and bacterivore networks under different long-term land uses including cropland, grassland, and bare land. Diazotroph community was characterized by high-throughput sequencing. The results indicated that land use type influenced the dominant genera of diazotrophs and shaped the occurrence of specific indicator diazotroph taxa. Co-existing pattern analysis of diazotrophs and bacterivores indicated that grassland converted from cropland increased the complexity of diazotroph and bacterivore network structure. The number of nodes for diazotrophs and bacterivores was higher in grassland than in cropland and bare land. Random forest analysis revealed that six bacterivore genera *Cephalobus, Protorhabditis, Acrobeloides, Mesorhabditis, Anaplectus*, and *Monhystera* had significant effects on diazotrophs. Bacterivores were found to have predominantly negative effects in bare land. Different bacterivores had differing effects with respect to driving changes in diazotroph community structure. Structural equation model showed that land use could control diazotroph community composition by altering soil properties and regulating abundance of bacterivores. These findings accordingly enhance our current understanding of mechanisms underlying the influence of land use patterns on diazotrophs from the perspective of soil food webs.

## Introduction

Land use change is among the most prominent of human-induced environmental pressures affecting ecosystem function (de Vries et al., 2012; da Silva et al., 2021). Agricultural soils in particular are considered prime examples of disturbed systems, in which the associated changes can influence microbial communities by altering steady-state environmental conditions (Rocca et al., 2018). Numerous studies have reported the impact of land use on microbial communities in both natural and disturbed environments. For example, land use has been found to affect microbial diversity (Sun et al., 2020), distribution (Woloszczyk et al., 2020), and function (Singh et al., 2020). To a large extent, soil microbial communities and their associated functions determine the productivity of ecosystems (van der Heijden et al., 2008). Most studies in this context have reported that changes in land use can significantly affect belowground microbial diversity and function. However, these studies have tended to focus primarily on meta-microbial communities (Sarto et al., 2020; Salazar et al., 2021), whereas relatively few studies have examined the effects of land use change on the communities of specific functional bacteria.

Diazotrophs are a group of bacteria widely distributed in soils, in which they play prominent roles in nitrogen cycling, notably with respect to biological nitrogen fixation, an important source of soil nitrogen in both natural and agricultural ecosystems (Cooper and Scherer, 2012). Diazotroph communities thus have a substantial influence on soil nitrogen pools and plant growth (Rodrigues et al., 2018). Numerous previous studies have focused on the activity and diversity of diazotrophs (Chen et al., 2019; Liang et al., 2019), as well as the factors affecting diazotroph communities, such as fertilization (Fan et al., 2019), plant species richness (Tu et al., 2016), and soil type (Han et al., 2019), and gaining an understanding of the impact of land use on diazotroph communities is of particular importance from the perspectives of maintaining soil biodiversity and ecosystem function.

As in any ecosystem, the organisms inhabiting soil do not exist in isolation, but interact directly and indirectly with multiple other soil-dwellers. Alterations in microbial communities and interactions can lead to changes in soil ecosystem function (Graham et al., 2017; Jiang et al., 2017), and in this regard, co-existing network analysis has recently gained prominence as a tool for gaining a better understanding of the potential interactions among soil microbes (Fuhrman, 2009; Faust and Raes, 2012). Network topological parameters have been used in soil microbial ecology research to examine microbial resilience to environmental disturbance (Heleno et al., 2012; Landi et al., 2018), visualize the patterns of community structure and responses of specific taxa or functional groups to agronomic practices (Barberan et al., 2012; Hartmann et al., 2014; Jiang et al., 2017), and identify the individual taxa that play pivotal roles in maintaining community stability (Agler et al., 2016; Hartman et al., 2018). Recent studies have shown that soils are characterized by distinct microbial networks under conventional and reduced tillage (Hartman et al., 2018), conventional and organic management (van der Heijden and Hartmann, 2016), and warm and cold climatic regimes (Tang et al., 2021). To date, however, little attention has focused on the effects of different land use patterns on the co-existing patterns in diazotroph communities.

Soil nematodes are an important component of the soil biota and considered to be the most abundant animals on earth (Bardgett and van der Putten, 2014). These nematodes are assigned to different trophic groups according to their feeding habits, such as bacterivores, fungivores, plant parasites, and predators/omnivores (Yeates et al., 1993). Bacterivores feed on bacteria and thereby potentially influence bacterial community composition and function. For example, bacterivorous nematodes have been found to increase the abundance and activity of alkaline phosphomonoesterase-producing bacteria (Jiang et al., 2017), and the abundance of ammonia-oxidizing bacteria (Xiao et al., 2010). Diazotrophs are important functional microbes in the soil of terrestrial ecosystems, and the diazotroph community structure directly affects the nitrogen-fixing function of soil ecosystems (Nag et al., 2020). To date, however, the interactions between bacterivores and diazotrophs remain poorly understood. If soil nematodes and diazotroph communities are indeed closely linked, it would thus be of particular interest to identify which nematodes are the key taxa shaping the community dynamics of diazotrophs under different land uses.

Accordingly, in this study we sought to characterize the diazotroph communities and food web structure of diazotrophs and bacterivores under different long-term land uses including cropland, grassland, and bare land. The primary objectives of the study were to (1) determine the effect of land use on diazotroph community composition; (2) identify which bacterivores are the key taxa influencing diazotroph community structure under different land uses; and (3) establish the impact of land use on the structure of diazotroph and bacterivore networks. We hypothesized that different types of land use would be associated with specific diazotroph communities, that bacterivores are important driving factors of diazotroph community and the structure of diazotroph and bacterivore networks is responsive to land use.

## Materials and methods

### Site description

The study was conducted at the Hailun National Field Observation and Research Station, which is located in a monsoon climate region of Northeast China (47°27′N, 126°55′E). Annual precipitation is approximately 500–600 mm, mean annual temperature is 1.5°C, and the cumulative temperature above 0°C ranges from 2,400 to 2,500°C-days. The soils in the region are typical black soil (Udic Mollisol). The field was covered by native prairie grasses before it was converted to cropland about 100 years ago. Due to the impact of cropland use and natural erosion on soil biodiversity and soil fertility, natural abandoned land and bare land uses were artificially set up to explore the changes of soil ecosystem functions after cropland ecological restoration and deep disturbance.

### Experimental design and soil sampling

We collected soil samples from land under three different uses: cropland, grassland, and bare land. Both grassland and bare land were formed after cropland had been abandoned. Cropland soils were collected under soybean cultivation phase from the land of a soybean [*Glycine max* (L.) Merr.]–maize (*Zea mays* L.)–wheat (*Triticum aestivum* L.) rotation, which was investigated in a long-term crop rotation experiment established in 1991. Each plot size was 11 m × 7 m. Basal N and P fertilizers were applied as urea and ammonium phosphate, respectively. Annual fertilizer application was 92.4 kg N ha^–1^ and 16.9 kg P ha^–1^ for wheat, 27.0 kg N ha^–1^ and 30.1 kg P ha^–1^ for soybean, and 65.5 kg N ha^–1^ and 30.1 kg P ha^–1^ for maize. An additional 65.5 kg N ha^–1^ was applied at the booting stage of maize. Details of the long-term experiments have been described in a previous publication (Qiao et al., 2015). Grassland and bare land soil samples were collected from experimental plots (4.2 m × 5 m) with three replicates of a long-term soil restoration and ecological research study established in 2003 on a converted cropland, the details of which have been described previously (Zhang et al., 2020). There were 300 m between cropland and grassland or bare land. The grassland site was allowed to return to its natural state with natural grass vegetation and during this transition received no fertilizer inputs or tillage. The bare land site was maintained free of vegetation by periodic shallow manual hoeing when weeds were visible. The cropland site was plowed each year to a depth of approximately 20 cm after crops were harvested.

Soil sampling was conducted in July and August, 2017. In each plot, topsoil down to a depth of 20 cm was collected using a manual soil coring tube (5 cm diameter), with a composite of samples taken from 10 collection points being considered a replicate. In cropland, large soil blocks surrounding soybean plants were removed by hand and soil from among the roots was collected. All soil samples were sieved through a 2-mm mesh to remove root debris and stones, divided into three parts, placed in a foam box containing ice bags, and brought back to the laboratory for nematode collection, soil DNA extraction and soil property analysis.

### Soil property analysis

Soil organic carbon (SOC) and Total nitrogen (TN) were analyzed by a VarioEL CHN elemental analyzer (Heraeus Elementar VarioEL, Hanau, Germany). Alkali-hydrolyzable nitrogen (AN) was determined by alkaline hydrolysis and diffusion. Soil pH was measured in water extracts using a pH meter. Soil moisture was measured by drying soil samples at 105°C to stable weight.

### Nematode extraction and identification

Nematodes were extracted from 100-g fresh soil for 48 h using the Baermann tray method modified from the Baermann funnel method. The extracted nematodes were preserved in 20-mL tubes. Nematode suspension from each soil sample was used for nematode identification under a microscope (Olympus BX43, Tokyo, Japan) to the genus level. The soil nematodes were assigned to four trophic groups: plant parasites (Pp), bacterivores (Ba), fungivores (Fu), and predators/omnivores (Po) (Yeates et al., 1993).

### Soil DNA extraction and diazotroph community analysis

Total soil DNA was extracted from 0.5 g of soil with an E.Z.N.A. Soil DNA Kit (OMEGA, Norcross, GA, United States) following the standard protocol. DNA extractions were done for each soil sample. The quantity and quality of mixed DNA were checked with a NanoDrop 2000 spectrophotometer (Thermo Scientific, Waltham, MA, United States). The *nifH* gene was amplified using primer pairs *nifH*-F (5′-AAAGGYGGWATCGGYAARTCCACCAC-3′) and *nifH*-R (5′-TTGTTSGCSGCRTACATSGCCATCAT-3′) (Rösch et al., 2002). After sequencing, raw fastq files were demultiplexed, quality-filtered by Trimmomatic and merged by FLASH. Operational taxonomic units (OTUs) were clustered with 97% similarity cutoff using UPARSE^1^ (version 7.1) and chimeric sequences were identified and removed using UCHIME. The taxonomy of each *nifH* gene sequence was analyzed by FunGene^2^ (version 9.6). Raw sequences of *nifH* gene have been submitted to NCBI Sequence Read Archive with Nos. PRJNA754795 and PRJNA754807.

### Quantitative real-time PCR

The abundance of bacteria (16S rRNA gene) and diazotrophs (*nifH* gene) were determined based on quantitative real-time PCR (qPCR) performed in 20-μL reaction volumes using primer pairs 515 F/907 R for the 16S rRNA gene (Biddle et al., 2008) and *nifH*-F/*nifH*-R for the *nifH* gene (Rösch et al., 2002). The qPCR amplification program used has been described by Pan et al. (2020). The two target genes were cloned into a pMD18-T plasmid. Target gene abundance was calculated using standard curves. The standard curves were obtained by serially diluting plasmids containing fragments of the 16S rRNA gene and *nifH* gene to final gene concentrations of 10^–1^–10^–6^ of those in the original plasmid. Data analysis was performed using ICycler software (Bio-Rad, Hercules, CA, United States).

### Identification of land use-indicator operational taxonomic units

Complementary approaches were used to identify the response of OTUs to the effect of land use on the soil diazotroph community. We initially employed Indicspecies (1.7.9) to identify indicator species (*p* < *0.05*) (de Cáceres et al., 2010; Hartman et al., 2018), and thereafter used STAMP (2.1.3) to determine the significantly enriched OTUs (*p* < *0.05*) of each treatment based on changes in relative abundance. OTUs detected using both approaches were considered to be indicator OTUs, which were visualized using graphical phylogenetic analysis (GraphlAn). Heat maps were constructed to indicate the indicator OTUs of each land use type, and the histogram in the outermost circle represents the indicated value of the indicator OTUs.

### Co-existing network

Two types of co-existing networks were constructed in this study, namely, a meta-network of diazotrophs in soil under all three land uses, and a network of diazotrophs and bacterivores for each treatment (land use type). Networks were constructed based on Spearman correlation (*r* > 0.7 and *p* < 0.05) using the R package igraph (1.2.5). The descriptive and topological properties of the networks were determined using Cytoscape (version 3.5.1), and the division of network modules was based on the greedy optimization of the modularity algorithm. Within-module connectivity (Zi) and among-module connectivity (Pi) were used to characterize the role of OTU in the network (Guimera and Amaral, 2005). All networks were visualized using Gephi software (0.9.2), in which nodes with a high value of either Zi or Pi were defined as keystone taxa, including module hubs (Zi > 0.25, Pi ≤ 0.62), connectors (Zi ≤ 0.25, Pi > 0.62), and network hubs (Zi > 0.25, Pi > 0.62). Network hubs, module hubs, and connectors were termed keystone network topological features, and these nodes were considered to have a significant impact on the stability and resistance of the network structure.

### Data analysis

Ordinary one-way ANOVA was used to identify significant changes in the relative abundance of diazotrophs at the phylum level (*p* < *0.05*). Non-metric multidimensional scaling (NMDS) based on a Bray–Curtis matrix was used to analyze changes in the composition of diazotrophs at the OTU level. In order to examine the effects of different land uses on diazotroph communities, we performed analysis of similarities (ANOSIM), non-parametric multivariate analysis of variance (adonis), and a multiple response permutation procedure (MRPP), all three methods of which were based on Bray–Curtis dissimilarities and carried out using the corresponding functions in the vegan (2.5-6) program in R (4.0.2). Pearson correlation analysis was used to identify relationships between the numbers of *nifH* gene copies. We used Factoextra (Ver. 1.0.7) to perform principal component analysis (PCA) on bacterivores at the genus level, and the randomForest (4.6-14) package was used to analyze the relationship between bacterivores and diazotrophs, and to determine those bacterivores that had a significant influence on diazotrophs (*p* < 0.05). Spearman correlation analysis was used to identify relationships between bacterivores and the indicator diazotroph OTUs. Structural equation modeling (SEM) was used to analyze the direct and indirect effects of soil property and community structure of bacterivores on diazotroph community properties. SEM was performed by the robust maximum likelihood estimation using AMOS 21.0 (Amos, Development Corporation, Meadville, PA, United States). Chi-square, *p*-value and goodness of fit index were used to evaluate the fitness of SEM (Hooper et al., 2008).

## Results

### Diazotroph community composition

**FIGURE 1:**
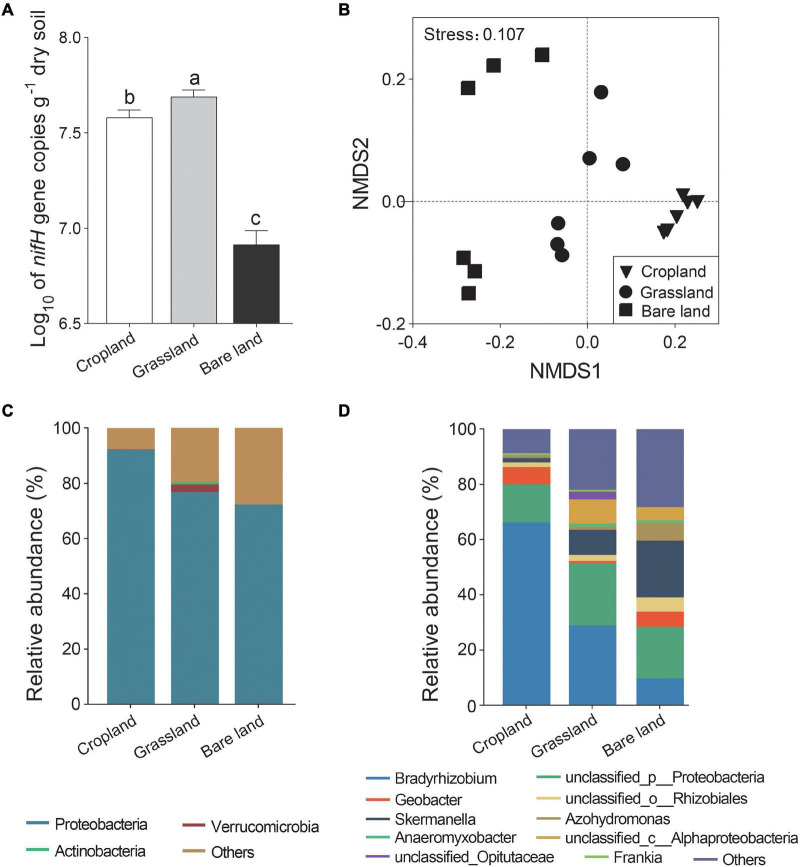
Abundance **(A)**, non-metric multidimensional scaling (NMDS) of *nifH* gene **(B)**, relative abundance of *nifH* gene at phylum **(C)**, and genus **(D)** levels. Different lowercase letters indicate significant differences among land uses at *p* < 0.05.

We found that abundance of the *nifH* gene varied significantly among the three land uses, being most abundant in grassland, followed by cropland, and lowest in bare land (Figure 1A, *p* < 0.05). Shannon index of diazotrophs was higher in cropland and bare land than that in grassland (Supplementary Figure 1, *p* < 0.05). Land use significantly altered the composition of the *nifH* gene community (Figure 1B and Supplementary Table 1, *p* < 0.001). At the OTU level, the *nifH* gene community developed in the direction of bare land, cropland, and grassland *via* axis 1 in the NMDS (Figure 1B). For all land use types, *Proteobacteria* was the predominant phyla (Figure 1C), and the relative abundance of *Verrucomicrobia* was highest in grassland. There were five, eight, and seven genera of diazotrophs with relative abundances higher than 1% in cropland, grassland, and bare land soils, respectively, accounting for 89.49, 76.51, and 70.84% of the relative abundance, respectively (Supplementary Table 2). *Bradyrhizobium* was the predominant bacterial genus in cropland and grassland soils, whereas *Skermanella* was predominant in bare land soil (Figure 1D). *Geobacter* accounted for 6.17 and 5.51% of relative abundance in cropland and bare land soils, respectively, but only 0.84% of relative abundance in grassland soil. The relative abundance of *Azohydromonas* was 6.51% in bare land, but only 0.63 and 1.16% in cropland and grassland soils, respectively. Although detected in the grassland soil, *Frankia* appeared to be absent from cropland and bare land soils.

A total of 84, 62, and 13 *nifH* gene indicator OTUs were detected in cropland, grassland, and bare land soils, respectively (Supplementary Table 3). At the phylum level, the *nifH* indicator OTUs belonged to Proteobacteria in cropland and bare land, and Proteobacteria, Actinobacteria, Verrucomicrobia, and Cyanobacteria in grassland (Supplementary Figure 2). Verrucomicrobia and Cyanobacteria were strongly indicative phyla in grassland. At the genus level, *Geobacter* and *Bradyrhizobium* were the strongest indicative genera in cropland, *Frankia* was the strongest indicative genus in grassland, and *Skermanella* was the most indicative in bare land.

### Land use effects on the co-existing patterns of diazotrophs

**FIGURE 2:**
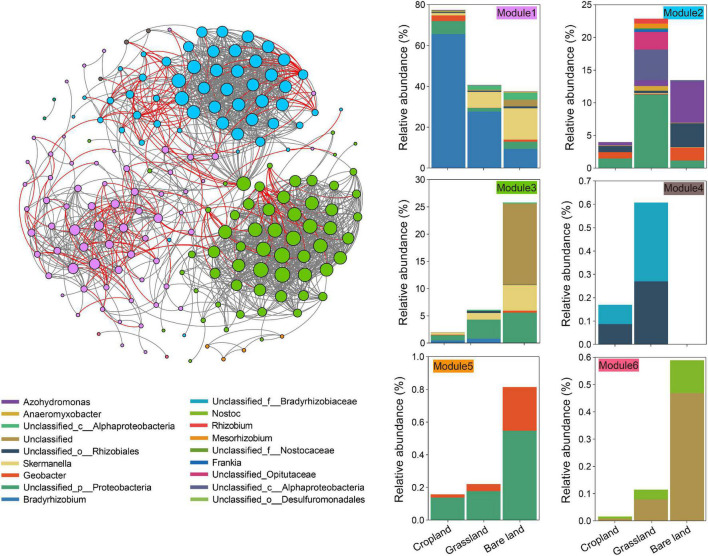
Co-existing patterns of the *nifH* gene. The links between two nodes represent a strong (*r* > 0.7) and significant (*p* < 0.001) correlation. Operational taxonomic units (OTUs) are colored by their association to the different modules. Cumulative relative abundance of *nifH* gene indicates the overall response of indicator modules to land use.

On the basis of our examination of the co-existing patterns of OTUs in the meta-network of diazotrophs (Figure 2), we established that abundance patterns of module associations responded to different land uses. In total, there were six modules in the meta-network (Figure 2), each of which comprised a taxonomically different set of diazotrophs. The soils under three land uses had a high relative abundance in module 1, which also contained OTUs specific to cropland soil, whereas modules 2 and 4 contained OTUs specific to grassland soil, and modules 3, 5, and 6 contained OTUs specific to bare land soil. We identified three keystone taxa, among which OTU2486 and OTU2587 were keystones in the module related to cropland, and OTU427 was the keystone in the module related to grassland (Supplementary Table 4).

### Effects of land use on soil nematode community structure

**FIGURE 3:**
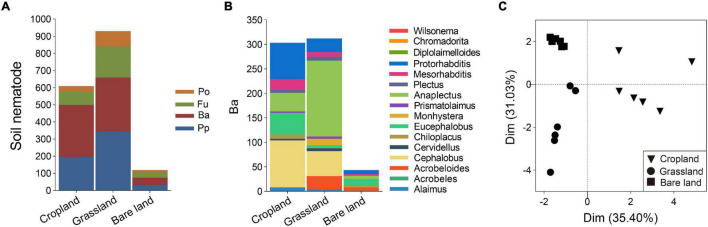
Abundance of soil nematode trophic groups **(A)**, abundance of bacterivores at genus level **(B)**, and principal component analysis (PCA) of bacterivores **(C)**. Abundance of soil nematodes is presented as individuals per 100 g dry soil. Pp, plant parasites; Ba, bacterivores; Fu, fungivores; Po, predators/omnivores.

Land use was also found to affect the composition of soil nematode community, with grassland soil being characterized by the highest nematode abundance at 929 individuals per 100 g dry soil, followed by cropland and bare land (Figure 3A). In terms of trophic groups, plant parasites and bacterivores were dominant in grassland soil, whereas bacterivores were predominant in cropland soil. With respect to bacterivores, *Cephalobus, Eucephalobus, Anaplectus*, and *Protorhabditis* were the dominant genera in cropland soil, *Cephalobus* and *Anaplectus* were dominant in grassland soil, and *Eucephalobus* was dominant in bare land soil (Figure 3B and Supplementary Figure 3). For the soil bacterivore community, PCA analysis revealed that cropland was separated from grassland and bare land *via* principal component 1, whereas grassland was separated from bare land *via* principal component 2 (Figure 3C).

### Effect of land use on the network structure of diazotrophs and bacterivores

**FIGURE 4:**
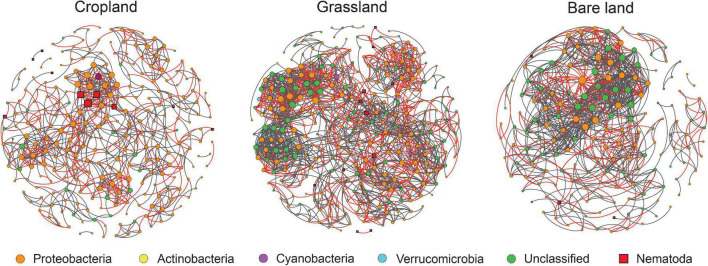
Networks between bacterivores and diazotrophs. For each panel, the size area of each node is proportional to the number of connections (degree). Gray and red lines represent positive and negative correlations between two nodes, respectively.

Network analysis was used to determine the co-existing patterns of diazotrophs and bacterivores in soils under different land uses. Overall, we found that land use has a clear effect on the networks of bacterivores and diazotrophs. Modularity was highest in cropland at 0.79, and lowest in bare land at 0.54 (Table 1). Bacterivores were observed to be more closely correlated with diazotrophs in cropland and grassland than in bare land (Figure 4 and Table 1). In the co-existing networks, grassland was characterized by a high connectivity between diazotrophs and bacterivores, as indicated by the higher number of diazotroph and bacterivore nodes (Table 1). The grassland network was found to contain the highest number of links, followed by that of bare land and cropland, while the ratio of negative to positive links increased from 0.38 to 0.69 from bare land to grassland and cropland (Table 1). The number of links between bacterivores and diazotrophs was found to be highest in cropland (60 links), followed by grassland (47), and least in bare land (9). Similarly, when expressed as a percentage of total links, the number of links between bacterivores and diazotrophs was highest in cropland (8.6%). Network connectivity was found to be highest in grassland, followed by bare land and cropland, and conversely, path length was greatest in cropland, followed by grassland and bare land. We identified 10 diazotroph keystone taxa that appear to be indicator to land use type (Supplementary Table 5): three (OTU2788, OTU2729, and OTU2714) for cropland, and seven (OTU2652, OTU1311, OTU347, OTU506, OTU203, OTU914, and OTU1117) for grassland. However, we detected no similar keystone taxa associated with bare land.

**TABLE 1:** Topological properties of networks between bacterivores and diazotrophs in different land uses.

Network metrics	Cropland	Grassland	Bare land
Modularity	0.79	0.72	0.54
Number of nodes for diazotrophs	253	297	169
Number of nodes for bacterivores	10	11	3
Number of links	702	1660	884
Number of positive links	416	1113	642
Number of negative links	286	547	242
Ratio of negative to positive links	0.69	0.49	0.38
Links between diazotrophs	637	1606	875
Links between bacterivores and diazotrophs	60	47	9
Connectivity	5.34	10.77	10.28
Network diameter	16	13	13
Average path length	6.80	5.23	4.02

### Effect of bacterivores on network structure and diazotroph community

**FIGURE 5:**
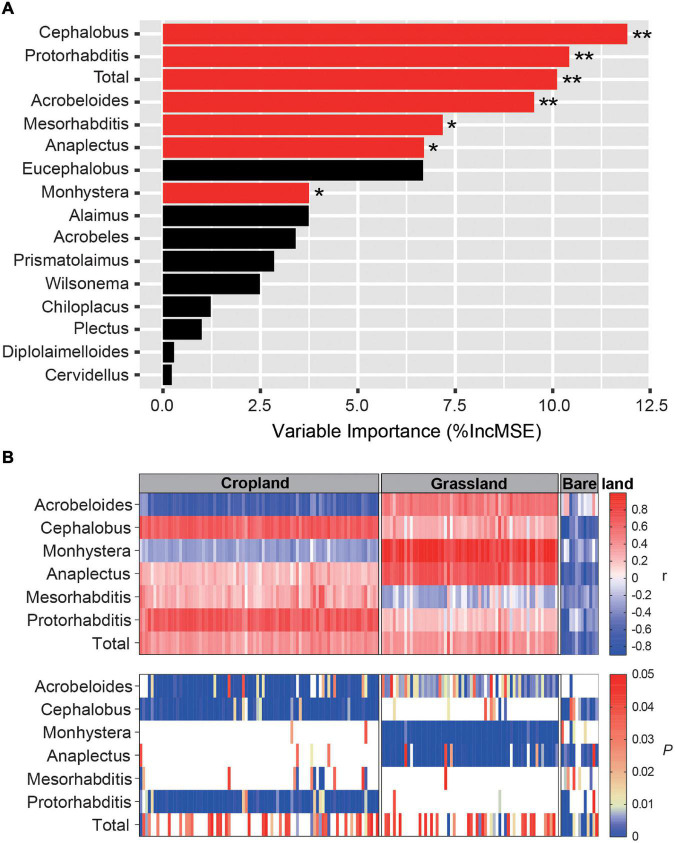
Random forest model output relating abundance of bacterivores to abundance of diazotrophs. Influence of bacterivores on diazotrophs **(A)** and correlation between main bacterivores and diazotrophs in different land uses **(B)**. The red and black bars in Panel **(A)** indicate that bacterivores have significant and unsignificant effects on diazotroph community composition, respectively. * and ** indicates that bacterivores have significant influence on diazotrophs at *p* < 0.05 and *p* < 0.01, respectively. *r* is the correlation coefficient. *P* indicates significance level. Ruler shows a maximum *p* value of 0.05. Blank in Panel **(B)** indicates that *p* > 0.05, and the correlation between main bacterivores and diazotrophs is not significant. The color markers indicate *p* < 0.05, indicating a significant correlation.

Pearson correlation analyses revealed that nematode species richness and abundance were significantly and positively correlated with the number of *nifH* gene copies and NMDS1 of the *nifH* gene (Supplementary Figure 4, *p* < 0.001). The PC1 of bacterivore PCA was significantly and negatively correlated with the network connections between nematodes and diazotrophs (*p* < 0.05). Using the random forest model to assess the effects of bacterivores on diazotrophs, we identified *Cephalobus, Protorhabditis, Acrobeloides, Mesorhabditis, Anaplectus*, and *Monhystera* to be the key bacterivore genera influencing diazotroph abundance (Figure 5A, *p* < 0.05, *p* < 0.01). We also examined the relationship between indicator OTUs and key bacterivores using Spearman correlation analysis (Figure 5B), which indicated that the following bacterivore genera have significant effects on diazotroph abundance in soil under different land uses: *Acrobeloides, Cephalobus*, and *Protorhabditis* in cropland; *Acrobeloides, Monhystera*, and *Anaplectus* in grassland; and *Cephalobus* and *Anaplectus* in bare land (Figure 5B). Moreover, we established that the direction of the influence of bacterivores on diazotrophs differed according to land use type (Figure 5B). *Acrobeloides* was found to have a significant negative effect on diazotroph abundance in cropland, although it had a positive effect in grassland, whereas *Monhystera* and *Anaplectus* had a significant positive effect in grassland but a negative effect in bare land. In bare land soil, the significant effects of bacterivores on diazotrophs were predominantly negative.

### Correlation among soil properties, community structure of bacterivores, and diazotroph community structure

**FIGURE 6:**
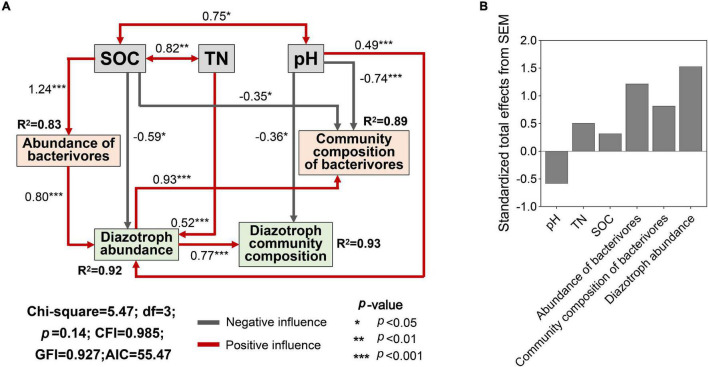
Structural equation model (SEM) of soil properties, community structure of bacterivores and diazotroph community structure **(A)** and Standardized total effects of each predictor on diazotroph community structure **(B)**. Community composition of bacterivores and diazotrophs is presented by PC1 (Figure 3) and NMDS1 (Figure 1). SOC, soil organic carbon; TN, total soil nitrogen. *R*^2^ values indicate the explained proportion of response variation by SEM.

Land use affected the contents of TN, SOC, AN, and soil pH (Supplementary Figure 5, *p* < 0.05). The key abiotic variables (SOC, TN, and soil pH) were selected for SEM and provided a successful fit. The SEM model showed that SOC significantly and negatively affected the diazotroph abundance and the community composition of bacterivores (Figure 6A, *p* < 0.05), while TN and pH significantly and positively affected the diazotroph abundance (*p* < 0.001). Soil pH had negative effect on the community composition of bacterivores and diazotrophs (*p* < 0.05, *p* < 0.001). SOC was positively correlated with abundance of bacterivores (*p* < 0.001), which had a positive effect on the diazotroph abundance (*p* < 0.001). Diazotroph abundance was positively correlated community composition of diazotrophs and bacterivores (*p* < 0.001). SEM showed that bacterivores contributed more effect on diazotroph community composition than individual soil properties (Figure 6B).

## Discussion

### Effect of land use on diazotroph communities

Our analyses of the influence of land use type on soil diazotroph communities revealed that these bacteria are characterized by differential responses to the three assessed land-use types. The grassland and bare land sites from which we collected soil were originally used for crop production and were converted to grassland and bare land in 2003. We detected differences in diazotroph community structure after the cropland had been converted to long-term land uses of grassland and bare land, with increases in diazotroph abundance detected in grassland, and reductions in abundance in bare land (Figure 1A). We speculate that the nutritional characteristics of soils under different land uses may contribute to promoting and maintaining differences in the abundance of specific diazotrophs. Moreover, differences in plant biomass in grassland and bare land may have an indirect effect on the abundance of diazotrophs; for example, the root exudates of plants are known to stimulate diazotroph growth and activity (Burgmann et al., 2005).

Previous studies in this field have tended to focus to a greater extent on the response of overall bacterial community structure to land use. In the present study, we found that land use has an appreciable influence on the structure of the soil diazotroph community. Diazotroph community structure of grassland and bare land developed from that of cropland to two opposite directions (Figure 1B). This is consistent with previous findings indicating that land-use conversion can alter soil microbial community composition and bacterial diversity (Rodrigues et al., 2013; de Carvalho et al., 2016), and is conceivably attributable to the strong influence of vegetation cover and type on diazotroph community structure. Lovell and Davis (2012) reported that diazotroph community structures in different vegetation zones are significantly different. Vegetation type has direct influence on microbial community, which affects the relative abundance and diversity of indigenous microbial communities at the root and in the rhizosphere (Fierer and Jackson, 2006; Berg and Smalla, 2009). This is likely due to different soil microenvironments produced by different plant species. Owing to their inability to adapt to environment change, certain diazotrophs can be eliminated or suppressed (Zhao et al., 2019), which could thus promote changes in community structure.

In the present study, we detected *Frankia* in grassland soil, but these bacteria could not be detected in cropland and bare land soils. This is likely due to the fact that *Frankia* are symbiotic bacteria that live in the root nodules of non-legume plants, so *Frankia* were detected in grassland but not in cropland with soybean and bare land without vegetation. Within soil ecosystems, dominant bacteria play an important role in maintaining soil ecological functions, and among the assessed soils, we found that *Bradyrhizobium* was the predominant genus in cropland and grassland soils, whereas bacteria in the genus *Skermanella* predominated in bare land soil (Figure 1D). It would seem likely that this difference reflects the influence of vegetation litter and root exudates on soil functional microbes (Somers et al., 2004; Wardle et al., 2004; Roberts et al., 2009), and indicates that the nitrogen-fixing function of the soil bacterial community is altered in response to land use change.

### Land use-indicator diazotrophs

We identified certain land use-indicator OTUs that can serve as indicator taxa to explain the β-diversity patterns associated with land use. For example, *Skermanella* was found to be the strongest indicator genus of bare land, which is consistent with the aforementioned distinction between bare land and the two other land use types. Furthermore, we found that the indicator genera for cropland (*Geobacter* and *Bradyrhizobium*) differed from the (*Frankia*) indicator for grassland (Supplementary Figure 2), which was also congruent with the marginal separation in β-diversity patterns. In response to the conversion from cropland to grassland and bare land, differences emerged in the indicator taxa profiles of soil under the different land uses, which can presumably be attributed to the dissimilar soil environments that have evolved under the different land uses, resulting in dissimilar selection pressures on diazotrophs. In this regard, a previous study has reported that *Geobacter* and *Bradyrhizobium* are widely distributed in cropped fields, and that their relative abundance is higher in non-compacted soils than in compacted soils (Fan et al., 2018). Another reason may be the predation and feeding preferences of bacterivores, which affect bacterial abundance (Yan et al., 2016; Irshad and Yergeau, 2018). In present study, land use affected the abundance of bacterivores with different feeding preferences. Our findings indicated that different land use types favor diazotrophs with different indicators.

### The co-existing network pattern of diazotrophs

Co-existing network analyses provide important insights into diazotroph community structure, as they highlight correlative phenomena that reflect the ecological interactions among taxa (e.g., mutualisms) (Faust and Raes, 2012; Weiss et al., 2016). In the meta-networks analysis in the present study, we identified modules containing a high relative abundance of OTUs responding similarly to different land uses, and noted that OTUs were also grouped into distinct modules reflecting the different land uses (Figure 2). The grouped diazotrophs respond in a similar manner to specific land uses, and thus cluster together. The finding that different modules were associated with different land uses would tend to indicate that land use affects the ecological cooperation of diazotrophs. Keystone taxa, for example, frequently interact with multiple other taxa and play ecologically important roles in determining community dynamics and function (Berry and Widder, 2014; Ma et al., 2016), and in the present study, we identified three keystone diazotroph OTUs that appear to be indicator for land use: two associated with cropland, and one with grassland. Such differences in keystone taxa among different land uses indicated that land use affects the nitrogen fixation function of soil microbiota (Fan et al., 2019).

### Land use effects on soil nematode community structure

We found that grassland soil had the highest abundance of soil nematodes, followed by cropland soil, with bare land soil being characterized by the lowest abundance (Figure 3A). These findings are consistent with those reported previously (Kandji et al., 2001; Pan et al., 2012) and is assumed to be associated with the considerably denser root network of the diverse plant species in grassland compared with that of cropland, which can support a more abundant soil biota. However, inconsistent observations in this regard have been reported in the literature. For example, Gao et al. (2021) found that nematode abundances were higher in disturbed croplands and orchards with external inputs than in undisturbed grass–shrub land without external inputs. Such disparate findings plausibly reflect differences in soil fertility. Gao et al. (2021) conducted their study in calcareous and red soils, which are notably poorer (less fertile) than the Mollisols examined in the present study. Mollisols are fertile soils, and even in the absence of fertilization, vegetation growing in the grasslands can develop normally and support rhizospheric soil biota.

Bacterivores are important predators that play a central role in regulating soil microbial community structure. In the present study, we identified the bacterivore *Eucephalobus* as the dominant nematode in cropland and bare land soils, and also found that the relative abundance of *Eucephalobus* in bare land soil was higher than that previously reported (Pan et al., 2012). We suspect that this dominance reflects the life history strategy of these nematodes with respect to coping with adverse environmental conditions, given that *Eucephalobus* belongs to the family Cephalobidae, the members of which are noted for their ability to survive in soil with both poor and abundant resources, and in unfavorable environments, such as those characterizing extremely arid regions (Nielsen et al., 2014). We found that land use had shaped the community structure of bacterivores, and in this regard, it was notable that the composition of bacterivores in cropland soil was separated from that in grassland and bare land soils *via* axis 1 in the PCA analysis (Figure 3C). This is consistent with a previous finding indicating that the community structure of soil nematodes in native vegetation differed from that of croplands (Wu et al., 2021). We suspect our observations in this regard can be explained in terms of changes in the physical and biological properties of soil linked to an increased input of organic material, diverse vegetation cover, and deposition of roots following the conversion from cropland to grassland (Scharroba et al., 2016; Haghverdi and Kooch, 2019). The structure of the bacterivore community inhabiting grassland soil was found to differ from those characterizing cropland and bare land soils, which is consistent with previous findings indicating that land use affects the structure of soil nematode communities (Pan et al., 2012; Siebert et al., 2020). Compared with cultivated cropland, grasslands typically support a greater diversity of plant species, and are characterized by a higher soil C input and denser root deposition, which would influence the structure of the soil microbial community, and consequently, the associated nematode predators (Ferris et al., 2001; Zhang et al., 2017). Our findings also indicated that in both cropland, with long-term tillage and fertilization, and bare land, with only shallow manual hoeing and no fertilization, there has been a degradation of bacterivore community structure.

### Effect of land use on the network structure of diazotrophs and bacterivores

We found that changes in land use have contributed to promoting changes in the structure and complexity of diazotroph and bacterivore networks. Our observations indicated that the regular cropping of land is associated with an overall reduction in the complexity of the network between diazotrophs and bacterivores, assessed in terms the connectivity and path length of co-existing networks. This also indicated that the structure of diazotroph and bacterivore networks is vulnerable to perturbations; i.e., the grassland soil with no tillage or fertilizer input tends to be less disturbed than cropland soil. Previous studies have reported that networks with greater connectivity are more robust to environmental perturbation (Landi et al., 2018; Shu et al., 2021), and that intense agricultural management can have the effect of reducing network connectivity and complexity (Banerjee et al., 2019).

Our co-existing network analysis revealed that the network characterizing grassland had the highest number of nodes for diazotrophs and bacterivores, and the largest number of links, thereby indicating that conversion to grassland has the effect of increasing the connectivity of diazotrophs, as well as the structural complexity of the diazotroph and bacterivore network. Although the network constructed for cropland had a higher number of nodes for diazotrophs than the network for bare land, it had a lower number of links and connectivity (Table 1), indicating that regularly cropping land reduces the cooperative interactions among diazotrophs and overall network stability. These differences are assumed to be attributable to the resource-limited conditions in bare land. The bare land examined in the present study had no vegetation cover and no soil nutrient input. Under these circumstances, it may be necessary for diazotrophs to enhance their mutual functional compensation and cooperation, which is important for survival in this environment. Consequently, microbial communities in resource-limited soils could have higher network complexity than those associated with land uses with higher inputs (Banerjee et al., 2019).

Given that food web stability is dependent on negative feedback, predator–prey interactions tend to be the dominant relationships among soil food web components. Although previous studies have often examined the effect of land use on the structures of soil microbe and nematode communities (Singh et al., 2020; Karuri, 2021), the present study is, to the best of our knowledge, the first to investigate the effect of land use on the interaction of soil nematodes and bacteria. By focusing on a specific functional group (diazotrophs), we have enhanced our current understanding of the ecological interactions between bacteria and bacterivores. We found that the number of network nodes for bacterivores was highest in grassland and lowest in bare land, whereas the proportion and number of links between bacterivores and diazotrophs was highest in cropland. These findings thus indicated that a change in land use can promote changes in the associations between bacterivores and diazotrophs, which were closer and stronger in cropland.

### Land use affects the interaction between diazotrophs and bacterivores

Our Pearson correlation analyses revealed that the structure of the bacterivore community influences that of the diazotroph community. We found that the PC1 of bacterivore PCA was significantly correlated with the network connections between bacterivores and diazotrophs, thereby indicating that the community composition of bacterivores influences the structural stability of the network. Random forest analysis was used to further examine the driving effect of bacterivores on diazotrophs inhabiting soil under different land use types. We established that six bacterivore genera have significant effects on these diazotrophs, and that these bacterivores have differential effects on diazotrophs, with *Acrobeloides* and *Monhystera* having negative effect on diazotroph abundance in cropland and bacterivores in the remaining four genera having positive effect (Figure 5B). These differences are assumed to reflect the selective feeding traits of bacterivores which adopt a predatory mode of feeding to maximize fitness (Bonkowski et al., 2009). When prey species are present in a soil ecosystem, bacterivores must coordinate their selective feeding traits to ensure long-term coexistence. In this regard, we also observed differences in the direction of influence of the same bacterivore genera under different land use types, such as *Acrobeloides* and *Mesorhabditis* in cropland and grassland, which could be attributable to differences in the community structure and species of diazotrophs in cropland and grassland.

Different bacteria have different responses or strategies with respect to predators. We thus speculate that the more negative effects of bacterivores on diazotrophs in bare land than in cropland and grassland could be associated with the lower abundance and unsuitability of diazotroph taxa available for nematode consumption in bare land. We found that the abundance of the *nifH* gene in bare land was lower than that in cropland and grassland. Conversely, however, predation by bacterivores may actually have a positive effect on their prey, given that predatory pressure can contribute to the dispersal of prey to new niches and increase their activity (Ingham et al., 1985; Jiang et al., 2017). This, nevertheless, is contingent on the presence of sufficient soil resources to support the reproduction of prey species, whereas the bare land soil examined in the present study is characterized by low nutrient levels. The bacteria inhabiting stressed soil environments can, however, also evolve different strategies (novel physical and chemical means) to evade nematode predation (Cressman and Garay, 2009; Jousset, 2011), and increase cooperation and species compensation as a defensive strategy to counter predatory pressure and resource constraints. Such responses may explain our finding that the conversion of cropland to bare land increased the connectivity between diazotrophs and bacterivores.

Structural equation modeling model was used to show the relationships among soil properties, bacterivore and diazotroph community structure, as well as the potential effect path of land use on the latter two. The key soil properties SOC, TN, and pH directly affected diazotroph abundance (Figure 6A), which was significantly correlated with diazotroph community composition, suggesting that land use can directly control diazotroph community structure *via* altering soil properties. This is consistent with previous findings that soil properties influence diazotroph diversity, especially TN and pH (Chen et al., 2019; Han et al., 2019). This may be because different habitats have different selectivity on diazotroph groups, and the main environmental factors controlling diazotroph communities are obviously habitat-dependent (Hayden et al., 2010). The abundance of bacterivores indirectly affected the diazotroph community composition *via* influencing the diazotroph abundance, and the overall effect of bacterivores on diazotroph community was larger than that of soil properties. These results suggested that bacterivores are important drivers regulating the community structure of diazotrophs, and more attention should be paid to the interaction between prey and predator under the circumstance of land use conversion. Collectively, our findings indicated that changes in land use can induce corresponding changes in the community structure of bacterivores and the interactions of bacterivores and diazotrophs, which, by controlling the community structure of diazotrophs, may play an important role in the regulation of nitrogen cycling.

## Conclusion

The findings of this study indicated that changes in land use are accompanied by changes in soil diazotroph abundance and community composition, and shape the profiles of specific indicator diazotrophs. The changes in land use also had the effect of altering the ecological cooperation of diazotrophs, as indicated by the different co-existing patterns of diazotrophs and the network structure of diazotrophs and bacterivores. Conversion from cropland to grassland was found to enhance the complexity of the diazotroph and bacterivore networks. The cultivation practices associated with cropland are believed to contribute to the cooperation of diazotrophs and promote a stronger interaction between bacterivores and diazotrophs. Bacterivores play a prominent role in influencing diazotroph community composition and the structure of diazotroph and bacterivore networks. Collectively, these findings indicated that land use patterns could change the structure of diazotroph communities by altering the community structure of bacterivores, and thus shape the food web structure of diazotrophs and bacterivores.

## Data availability statement

The datasets presented in this study can be found in online repositories. The names of the repository/repositories and accession number(s) can be found in the article/Supplementary Material.

## Author contributions

FP and XZH designed the study. ZZ and HL performed the molecular analyses. ZZ, FP, and NM wrote the manuscript. JY and WZ provided funding support. XXH analyzed the soil properties. All authors contributed to the article and approved the submitted version.

## Conflict of interest

The authors declare that the research was conducted in the absence of any commercial or financial relationships that could be construed as a potential conflict of interest.

## Publisher’s note

All claims expressed in this article are solely those of the authors and do not necessarily represent those of their affiliated organizations, or those of the publisher, the editors and the reviewers. Any product that may be evaluated in this article, or claim that may be made by its manufacturer, is not guaranteed or endorsed by the publisher.

## Footnotes

1
http://drive5.com/uparse/

2
http://www.fungene-db.fr/
