# Supplementary material for: Land use alters diazotroph community structure by regulating bacterivores in Mollisols in Northeast China
Source: Front Microbiol. 2022 Jul 15;13:941170. doi: 10.3389/fmicb.2022.941170 (PMC9335130; doi:10.3389/fmicb.2022.941170)
Supplement: Supplementary file 1 [file Data_Sheet_1.DOCX]

**Supplementary information**

*Zhiming Zhang, Xiaozeng Ha^1^, Fengjuan Pan, Hang Liu, Jun Yan, Wenxiu Zou, Neil B. McLaughlin, Xiangxiang Hao.* *Land use alters the food web structure of diazotrophs and bacterivores in Mollisols in Northeast China.*

**Supplementary Table 1.** Statistics and probability of effect of land use on *nifH* gene compositions with three different statistical approaches.

|  | **Adonis** | |  | **ANOSIM** | |  | **MRPP** | |
| --- | --- | --- | --- | --- | --- | --- | --- | --- |
|  | F | *P* |  | R | *P* |  | δ | *P* |
| *nifH* gene | 0.257 | 0.001 |  | 0.842 | 0.001 |  | 0.375 | 0.001 |

Adonis, permutational multivariate analysis; ANOSIM, analysis of similarities, and MRPP, multi response permutation procedure.

**Supplementary Table** **2.** Relative abundances of diazotrophs under different land uses.

| **Phylum** | **Class** | **Order** | **Family** | **Genus** | **Cropland** | **Grassland** | **Bare land** |
| --- | --- | --- | --- | --- | --- | --- | --- |
| Proteobacteria | Alphaproteobacteria | Rhizobiales | Bradyrhizobiaceae | *Bradyrhizobium* | **66.29±0.08 a** | **29.01±0.08 b** | **9.76±0.06 c** |
| Proteobacteria | unclassified_p__Proteobacteria | unclassified_p__Proteobacteria | unclassified_p__Proteobacteria | unclassified_p__Proteobacteria | **13.75±0.03 c** | **22.40±0.04 a** | **18.64±0.08 b** |
| Proteobacteria | Deltaproteobacteria | Desulfuromonadales | Geobacteraceae | *Geobacter* | **6.17±0.02** a | 0.84±0.01 **c** | **5.51±0.04 b** |
| Proteobacteria | Alphaproteobacteria | Rhizobiales | unclassified_o__Rhizobiales | unclassified_o__Rhizobiales | **1.73±0.00 c** | **2.18±0.01 b** | **5.20±0.03 a** |
| Proteobacteria | Alphaproteobacteria | Rhodospirillales | Rhodospirillaceae | *Skermanella* | **1.56±0.01 c** | **9.10±0.02 b** | **20.44±0.04 a** |
| Proteobacteria | Betaproteobacteria | Burkholderiales | Alcaligenaceae | *Azohydromonas* | 0.63±0.00 **c** | **1.16±0.01 b** | **6.51±0.06 a** |
| Proteobacteria | Deltaproteobacteria | Myxococcales | Myxococcaceae | *Anaeromyxobacter* | 0.61±0.00 **c** | **1.04±0.01** a | 0.89±0.01 b |
| Proteobacteria | Alphaproteobacteria | unclassified_c__Alphaproteobacteria | unclassified_c__Alphaproteobacteria | unclassified_c__Alphaproteobacteria | 0.51±0.00 **c** | **8.80±0.02 a** | **4.78±0.03 b** |
| Verrucomicrobia | Opitutae | Opitutales | Opitutaceae | unclassified_Opitutaceae | 0.04±0.00 **b** | **2.82±0.02** a | 0.00±0.00 b |
| Actinobacteria | Actinobacteria | Actinomycetales | Frankiaceae | *Frankia* | 0.00±0.00 b | 0.70±0.00 a | 0.00±0.00 b |

The values represent with averages and standard errors. Bold font represents dominant genus with relative abundance > 1%. Different lowercase letters indicate significant differences among land uses at p < 0.05.

**Supplementary Table 3.** Indicator OTU of different land uses.

|  | **Genus** | **Indval** | **Cropland** | **Grassland** | **Bare land** |
| --- | --- | --- | --- | --- | --- |
| OTU2673 | Bradyrhizobium | 0.797 | 65.59 | 27.31 | 9.15 |
| OTU2766 | Bradyrhizobium | 0.722 | 0.07 | 0.02 | 0.01 |
| OTU49 | unclassified_o__Rhizobiales | 0.696 | 0.02 | 0.00 | 0.00 |
| OTU2729 | unclassified_c__Alphaproteobacteria | 0.781 | 0.06 | 0.00 | 0.00 |
| OTU2613 | Azoarcus | 0.772 | 0.01 | 0.00 | 0.00 |
| OTU2442 | Geobacter | 0.886 | 0.39 | 0.00 | 0.01 |
| OTU2444 | Geobacter | 0.843 | 0.05 | 0.00 | 0.00 |
| OTU2470 | Geobacter | 0.702 | 0.05 | 0.00 | 0.00 |
| OTU2474 | Geobacter | 0.728 | 0.02 | 0.00 | 0.00 |
| OTU2485 | Geobacter | 0.877 | 0.53 | 0.03 | 0.01 |
| OTU2486 | Geobacter | 0.826 | 0.22 | 0.00 | 0.00 |
| OTU2502 | Geobacter | 0.810 | 0.02 | 0.00 | 0.00 |
| OTU2503 | Geobacter | 0.907 | 0.19 | 0.00 | 0.00 |
| OTU2525 | Geobacter | 0.794 | 0.02 | 0.00 | 0.00 |
| OTU2526 | Geobacter | 0.753 | 0.05 | 0.00 | 0.00 |
| OTU2529 | Geobacter | 0.955 | 0.09 | 0.00 | 0.00 |
| OTU2541 | Geobacter | 0.875 | 0.10 | 0.00 | 0.00 |
| OTU2548 | Geobacter | 0.858 | 0.15 | 0.00 | 0.00 |
| OTU2549 | Geobacter | 0.814 | 0.11 | 0.00 | 0.00 |
| OTU2578 | Geobacter | 0.826 | 0.05 | 0.00 | 0.00 |
| OTU2586 | Geobacter | 0.775 | 0.02 | 0.00 | 0.00 |
| OTU2592 | Geobacter | 0.826 | 0.03 | 0.00 | 0.00 |
| OTU2634 | Geobacter | 0.727 | 0.07 | 0.00 | 0.00 |
| OTU2754 | Geobacter | 0.840 | 0.02 | 0.00 | 0.00 |
| OTU2757 | Geobacter | 0.815 | 0.04 | 0.00 | 0.00 |
| OTU2760 | Geobacter | 0.941 | 0.07 | 0.00 | 0.00 |
| OTU2776 | Geobacter | 0.738 | 0.03 | 0.00 | 0.00 |
| OTU2799 | Geobacter | 0.954 | 0.08 | 0.00 | 0.00 |
| OTU2801 | Geobacter | 0.893 | 0.04 | 0.00 | 0.00 |
| OTU2803 | Geobacter | 0.813 | 0.03 | 0.00 | 0.00 |
| OTU2816 | Geobacter | 0.909 | 0.07 | 0.00 | 0.00 |
| OTU2822 | Geobacter | 0.827 | 0.91 | 0.00 | 0.00 |
| OTU2824 | Geobacter | 0.854 | 0.13 | 0.00 | 0.00 |
| OTU1282 | unclassified_o__Desulfuromonadales | 0.804 | 0.02 | 0.00 | 0.00 |
| OTU2543 | unclassified_o__Desulfuromonadales | 0.769 | 0.17 | 0.02 | 0.00 |
| OTU2591 | unclassified_o__Desulfuromonadales | 0.775 | 0.05 | 0.00 | 0.00 |
| OTU2563 | Anaeromyxobacter | 0.685 | 0.05 | 0.00 | 0.00 |
| OTU542 | Anaeromyxobacter | 0.746 | 0.18 | 0.01 | 0.05 |
| OTU2448 | unclassified_c__Deltaproteobacteria | 0.813 | 0.14 | 0.00 | 0.00 |
| OTU2538 | unclassified_c__Deltaproteobacteria | 0.775 | 0.02 | 0.00 | 0.00 |
| OTU2821 | unclassified_c__Deltaproteobacteria | 0.711 | 0.03 | 0.00 | 0.00 |
| OTU1060 | unclassified_p__Proteobacteria | 0.969 | 0.21 | 0.00 | 0.01 |
| OTU1200 | unclassified_p__Proteobacteria | 0.802 | 0.29 | 0.00 | 0.01 |
| OTU121 | unclassified_p__Proteobacteria | 0.808 | 0.03 | 0.00 | 0.00 |
| OTU2445 | unclassified_p__Proteobacteria | 0.944 | 0.04 | 0.00 | 0.00 |
| OTU2457 | unclassified_p__Proteobacteria | 0.740 | 0.08 | 0.00 | 0.00 |
| OTU2467 | unclassified_p__Proteobacteria | 0.850 | 0.07 | 0.00 | 0.00 |
| OTU2473 | unclassified_p__Proteobacteria | 0.760 | 0.03 | 0.00 | 0.00 |
| OTU2479 | unclassified_p__Proteobacteria | 0.907 | 0.07 | 0.00 | 0.00 |
| OTU2480 | unclassified_p__Proteobacteria | 0.744 | 0.05 | 0.00 | 0.00 |
| OTU2532 | unclassified_p__Proteobacteria | 0.718 | 0.02 | 0.00 | 0.00 |
| OTU2542 | unclassified_p__Proteobacteria | 0.900 | 0.12 | 0.00 | 0.00 |
| OTU2546 | unclassified_p__Proteobacteria | 0.907 | 0.05 | 0.00 | 0.00 |
| OTU2560 | unclassified_p__Proteobacteria | 0.877 | 0.13 | 0.00 | 0.00 |
| OTU2568 | unclassified_p__Proteobacteria | 0.694 | 0.06 | 0.00 | 0.00 |
| OTU2574 | unclassified_p__Proteobacteria | 0.828 | 0.33 | 0.09 | 0.00 |
| OTU2579 | unclassified_p__Proteobacteria | 0.738 | 0.05 | 0.00 | 0.00 |
| OTU2587 | unclassified_p__Proteobacteria | 0.986 | 0.29 | 0.00 | 0.00 |
| OTU2588 | unclassified_p__Proteobacteria | 0.722 | 0.03 | 0.00 | 0.00 |
| OTU2590 | unclassified_p__Proteobacteria | 0.958 | 0.17 | 0.00 | 0.00 |
| OTU2615 | unclassified_p__Proteobacteria | 0.781 | 0.13 | 0.05 | 0.00 |
| OTU2626 | unclassified_p__Proteobacteria | 0.727 | 0.13 | 0.03 | 0.00 |
| OTU2627 | unclassified_p__Proteobacteria | 0.840 | 0.05 | 0.00 | 0.00 |
| OTU2629 | unclassified_p__Proteobacteria | 0.838 | 2.08 | 0.04 | 0.00 |
| OTU2631 | unclassified_p__Proteobacteria | 0.799 | 0.32 | 0.01 | 0.00 |
| OTU2640 | unclassified_p__Proteobacteria | 0.712 | 0.06 | 0.00 | 0.00 |
| OTU2657 | unclassified_p__Proteobacteria | 0.694 | 0.09 | 0.01 | 0.00 |
| OTU2696 | unclassified_p__Proteobacteria | 0.670 | 0.02 | 0.00 | 0.00 |
| OTU2747 | unclassified_p__Proteobacteria | 0.827 | 0.05 | 0.00 | 0.00 |
| OTU2748 | unclassified_p__Proteobacteria | 0.696 | 0.02 | 0.00 | 0.00 |
| OTU2762 | unclassified_p__Proteobacteria | 0.699 | 0.03 | 0.00 | 0.00 |
| OTU2808 | unclassified_p__Proteobacteria | 0.831 | 0.18 | 0.00 | 0.00 |
| OTU2815 | unclassified_p__Proteobacteria | 0.878 | 0.04 | 0.00 | 0.00 |
| OTU2825 | unclassified_p__Proteobacteria | 0.938 | 0.23 | 0.00 | 0.01 |
| OTU2826 | unclassified_p__Proteobacteria | 0.676 | 0.03 | 0.00 | 0.00 |
| OTU2835 | unclassified_p__Proteobacteria | 0.862 | 0.02 | 0.00 | 0.00 |
| OTU2569 | unclassified_k__norank_d__Bacteria | 0.736 | 0.02 | 0.00 | 0.00 |
| OTU2614 | unclassified_k__norank_d__Bacteria | 0.896 | 0.04 | 0.00 | 0.00 |
| OTU2616 | unclassified_k__norank_d__Bacteria | 0.733 | 0.04 | 0.00 | 0.00 |
| OTU2621 | unclassified_k__norank_d__Bacteria | 0.797 | 0.17 | 0.00 | 0.00 |
| OTU2628 | unclassified_k__norank_d__Bacteria | 0.712 | 0.15 | 0.00 | 0.00 |
| OTU2635 | unclassified_k__norank_d__Bacteria | 0.699 | 0.05 | 0.00 | 0.00 |
| OTU2694 | unclassified_k__norank_d__Bacteria | 0.889 | 0.02 | 0.00 | 0.00 |
| OTU2843 | unclassified_k__norank_d__Bacteria | 0.734 | 0.03 | 0.00 | 0.00 |
| OTU405 | Frankia | 0.842 | 0.00 | 0.03 | 0.00 |
| OTU514 | Frankia | 0.807 | 0.00 | 0.42 | 0.00 |
| OTU421 | unclassified_f__Nostocaceae | 0.728 | 0.00 | 0.20 | 0.00 |
| OTU371 | unclassified_f__Bradyrhizobiaceae | 0.805 | 0.00 | 0.09 | 0.00 |
| OTU427 | unclassified_f__Bradyrhizobiaceae | 0.718 | 0.03 | 0.18 | 0.00 |
| OTU471 | unclassified_f__Bradyrhizobiaceae | 0.715 | 0.00 | 0.10 | 0.00 |
| OTU430 | Mesorhizobium | 0.686 | 0.00 | 0.68 | 0.00 |
| OTU302 | Rhizobium | 0.792 | 0.00 | 0.77 | 0.00 |
| OTU2646 | unclassified_o__Rhizobiales | 0.721 | 0.02 | 0.39 | 0.07 |
| OTU475 | unclassified_o__Rhizobiales | 0.789 | 0.00 | 0.03 | 0.00 |
| OTU1004 | unclassified_c__Alphaproteobacteria | 0.712 | 0.00 | 0.05 | 0.00 |
| OTU150 | unclassified_c__Alphaproteobacteria | 0.853 | 0.00 | 0.08 | 0.00 |
| OTU222 | unclassified_c__Alphaproteobacteria | 0.809 | 0.00 | 0.05 | 0.00 |
| OTU234 | unclassified_c__Alphaproteobacteria | 0.769 | 0.00 | 0.29 | 0.00 |
| OTU259 | unclassified_c__Alphaproteobacteria | 0.912 | 0.00 | 0.70 | 0.00 |
| OTU332 | unclassified_c__Alphaproteobacteria | 0.745 | 0.00 | 0.77 | 0.00 |
| OTU377 | unclassified_c__Alphaproteobacteria | 0.901 | 0.00 | 0.24 | 0.00 |
| OTU408 | unclassified_c__Alphaproteobacteria | 0.793 | 0.00 | 0.50 | 0.00 |
| OTU410 | unclassified_c__Alphaproteobacteria | 0.831 | 0.00 | 0.14 | 0.00 |
| OTU443 | unclassified_c__Alphaproteobacteria | 0.869 | 0.00 | 2.30 | 0.00 |
| OTU448 | unclassified_c__Alphaproteobacteria | 0.679 | 0.00 | 0.12 | 0.00 |
| OTU464 | unclassified_c__Alphaproteobacteria | 0.718 | 0.00 | 0.14 | 0.00 |
| OTU813 | unclassified_c__Alphaproteobacteria | 0.727 | 0.00 | 0.06 | 0.01 |
| OTU564 | Azohydromonas | 0.762 | 0.10 | 0.77 | 0.01 |
| OTU113 | Anaeromyxobacter | 0.674 | 0.00 | 0.02 | 0.00 |
| OTU866 | Anaeromyxobacter | 0.744 | 0.10 | 0.48 | 0.05 |
| OTU203 | unclassified_p__Proteobacteria | 0.827 | 0.00 | 0.06 | 0.00 |
| OTU270 | unclassified_p__Proteobacteria | 0.878 | 0.00 | 0.17 | 0.00 |
| OTU288 | unclassified_p__Proteobacteria | 0.747 | 0.00 | 0.13 | 0.00 |
| OTU303 | unclassified_p__Proteobacteria | 0.875 | 0.00 | 0.37 | 0.00 |
| OTU351 | unclassified_p__Proteobacteria | 0.873 | 0.00 | 0.30 | 0.00 |
| OTU355 | unclassified_p__Proteobacteria | 0.655 | 0.00 | 0.02 | 0.00 |
| OTU387 | unclassified_p__Proteobacteria | 0.775 | 0.00 | 0.06 | 0.00 |
| OTU396 | unclassified_p__Proteobacteria | 0.711 | 0.00 | 0.32 | 0.00 |
| OTU412 | unclassified_p__Proteobacteria | 0.867 | 0.03 | 0.99 | 0.00 |
| OTU418 | unclassified_p__Proteobacteria | 0.707 | 0.00 | 0.42 | 0.00 |
| OTU419 | unclassified_p__Proteobacteria | 0.860 | 0.01 | 0.14 | 0.00 |
| OTU429 | unclassified_p__Proteobacteria | 0.836 | 0.00 | 0.86 | 0.00 |
| OTU433 | unclassified_p__Proteobacteria | 0.899 | 0.20 | 1.53 | 0.04 |
| OTU441 | unclassified_p__Proteobacteria | 0.744 | 0.08 | 0.60 | 0.00 |
| OTU447 | unclassified_p__Proteobacteria | 0.897 | 0.00 | 0.61 | 0.00 |
| OTU459 | unclassified_p__Proteobacteria | 0.744 | 0.35 | 0.76 | 0.00 |
| OTU467 | unclassified_p__Proteobacteria | 0.727 | 0.00 | 0.05 | 0.00 |
| OTU529 | unclassified_p__Proteobacteria | 0.813 | 0.02 | 0.54 | 0.00 |
| OTU80 | unclassified_p__Proteobacteria | 0.684 | 0.00 | 0.04 | 0.00 |
| OTU1391 | unclassified_k__norank_d__Bacteria | 0.761 | 0.00 | 0.06 | 0.01 |
| OTU166 | unclassified_k__norank_d__Bacteria | 0.857 | 0.00 | 0.05 | 0.00 |
| OTU1876 | unclassified_k__norank_d__Bacteria | 0.760 | 0.00 | 0.01 | 0.00 |
| OTU191 | unclassified_k__norank_d__Bacteria | 0.728 | 0.00 | 0.02 | 0.00 |
| OTU2219 | unclassified_k__norank_d__Bacteria | 0.770 | 0.00 | 0.02 | 0.00 |
| OTU251 | unclassified_k__norank_d__Bacteria | 0.675 | 0.00 | 0.06 | 0.00 |
| OTU400 | unclassified_k__norank_d__Bacteria | 0.734 | 0.00 | 1.01 | 0.01 |
| OTU403 | unclassified_k__norank_d__Bacteria | 0.794 | 0.00 | 0.24 | 0.00 |
| OTU438 | unclassified_k__norank_d__Bacteria | 0.704 | 0.00 | 0.17 | 0.00 |
| OTU440 | unclassified_k__norank_d__Bacteria | 0.731 | 0.01 | 0.85 | 0.00 |
| OTU460 | unclassified_k__norank_d__Bacteria | 0.687 | 0.00 | 0.05 | 0.00 |
| OTU498 | unclassified_k__norank_d__Bacteria | 0.740 | 0.00 | 0.02 | 0.00 |
| OTU516 | unclassified_k__norank_d__Bacteria | 0.926 | 0.00 | 0.03 | 0.00 |
| OTU249 | unclassified_Opitutaceae | 0.843 | 0.00 | 1.84 | 0.00 |
| OTU508 | unclassified_Opitutaceae | 0.799 | 0.00 | 0.07 | 0.00 |
| OTU510 | unclassified_Opitutaceae | 0.796 | 0.00 | 0.60 | 0.00 |
| OTU2658 | unclassified_d__Unclassified | 0.704 | 0.00 | 0.05 | 0.00 |
| OTU1856 | Frankia | 0.808 | 0.00 | 0.00 | 0.00 |
| OTU2243 | Frankia | 0.740 | 0.00 | 0.00 | 0.00 |
| OTU1672 | Bradyrhizobium | 0.708 | 0.00 | 0.01 | 0.01 |
| OTU2109 | Rhizobium | 0.688 | 0.00 | 0.00 | 0.00 |
| OTU1663 | unclassified_c__Alphaproteobacteria | 0.921 | 0.00 | 0.00 | 0.00 |
| OTU308 | unclassified_c__Alphaproteobacteria | 0.716 | 0.01 | 0.01 | 0.01 |
| OTU530 | Anaeromyxobacter | 0.817 | 0.00 | 0.02 | 0.00 |
| OTU1664 | unclassified_p__Proteobacteria | 0.725 | 0.00 | 0.00 | 0.00 |
| OTU2086 | unclassified_p__Proteobacteria | 0.763 | 0.00 | 0.01 | 0.00 |
| OTU477 | unclassified_p__Proteobacteria | 0.722 | 0.00 | 0.02 | 0.02 |
| OTU960 | unclassified_p__Proteobacteria | 0.810 | 0.00 | 0.00 | 0.02 |
| OTU1678 | unclassified_k__norank_d__Bacteria | 0.748 | 0.00 | 0.00 | 0.00 |
| OTU1735 | unclassified_k__norank_d__Bacteria | 0.866 | 0.00 | 0.01 | 0.01 |
| OTU1787 | unclassified_k__norank_d__Bacteria | 0.770 | 0.00 | 0.00 | 0.00 |
| OTU1984 | unclassified_k__norank_d__Bacteria | 0.743 | 0.00 | 0.00 | 0.03 |
| OTU2123 | unclassified_k__norank_d__Bacteria | 0.827 | 0.00 | 0.00 | 0.00 |
| OTU2240 | unclassified_k__norank_d__Bacteria | 0.738 | 0.00 | 0.00 | 0.01 |
| OTU2260 | unclassified_k__norank_d__Bacteria | 0.801 | 0.01 | 0.00 | 0.00 |
| OTU2263 | unclassified_k__norank_d__Bacteria | 0.778 | 0.00 | 0.00 | 0.00 |
| OTU1679 | unclassified_d__Unclassified | 0.754 | 0.00 | 0.01 | 0.01 |
| OTU858 | unclassified_o__Rhizobiales | 0.697 | 0.00 | 0.00 | 0.07 |
| OTU1749 | Skermanella | 0.896 | 0.37 | 2.07 | 6.73 |
| OTU573 | unclassified_c__Alphaproteobacteria | 0.807 | 0.02 | 0.12 | 0.72 |
| OTU1315 | Azohydromonas | 0.745 | 0.20 | 0.00 | 3.60 |
| OTU1459 | Azohydromonas | 0.868 | 0.04 | 0.01 | 0.52 |
| OTU1412 | Geobacter | 0.713 | 0.10 | 0.03 | 0.42 |
| OTU1093 | Anaeromyxobacter | 0.669 | 0.01 | 0.00 | 0.03 |
| OTU1110 | unclassified_p__Proteobacteria | 0.858 | 0.02 | 0.05 | 0.29 |
| OTU1517 | unclassified_p__Proteobacteria | 0.860 | 0.00 | 0.00 | 1.64 |
| OTU1335 | unclassified_k__norank_d__Bacteria | 0.702 | 0.01 | 0.00 | 0.25 |
| OTU1474 | unclassified_k__norank_d__Bacteria | 0.731 | 0.00 | 0.00 | 0.08 |
| OTU869 | unclassified_k__norank_d__Bacteria | 0.705 | 0.00 | 0.02 | 0.25 |
| OTU1417 | unclassified_d__Unclassified | 0.770 | 0.00 | 0.00 | 0.05 |

**Supplementary Tabl**e **4.** Keystones of network of diazotrophs under different land uses.

| **Treatment** | **Nodes_id** | **Within_module_connectivities** | **Among_module_connectivities** | **Degree** | **Modularity** |
| --- | --- | --- | --- | --- | --- |
| Cropland | OTU2486 | 2.60 | 0 | 26 | 1 |
|  | OTU2587 | 2.60 | 0 | 26 | 1 |
| Grassland | OTU427 | 0 | 0.64 | 5 | 4 |

**Supplementary Table** **5.** Keystones of network of diazotrophs and bacterivores under different land uses.

| **Treatments** | **Nodes_id** | **Within_module_connectivities(Zi)** | **Among_module_connectivities(Pi)** | **Degree** | **Genus** |
| --- | --- | --- | --- | --- | --- |
| Cropland | OTU2788 | -1.32 | 0.67 | 3 | unclassified_p__Proteobacteria |
|  | OTU2729 | -0.58 | 0.64 | 10 | unclassified_c__Alphaproteobacteria |
|  | OTU2714 | -1.41 | 0.64 | 5 | unclassified_p__Proteobacteria |
|  |  |  |  |  |  |
| Grassland | OTU2652 | 2.52 | 0 | 12 | unclassified_k__norank_d__Bacteria |
|  | OTU1311 | -1.03 | 0.69 | 8 | unclassified_k__norank_d__Bacteria |
|  | OTU347 | -1.24 | 0.68 | 11 | unclassified_k__norank_d__Bacteria |
|  | OTU506 | -0.71 | 0.66 | 18 | unclassified_p__Proteobacteria |
|  | OTU203 | -1.17 | 0.64 | 13 | unclassified_p__Proteobacteria |
|  | OTU914 | -1.43 | 0.63 | 4 | Geobacter |
|  | OTU1117 | -1.69 | 0.63 | 4 | unclassified_c__Alphaproteobacteria |

**Supplementary Figure 1.**


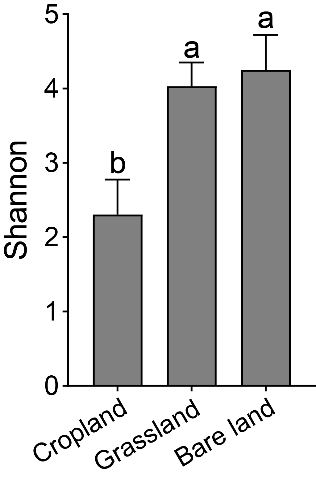


**Supplementary Figure 1.** Shannon index of diazotrophs in different land uses. Different lowercase letters indicate significant differences among land uses at *p* < 0.05.

**Supplementary Figure 2.**


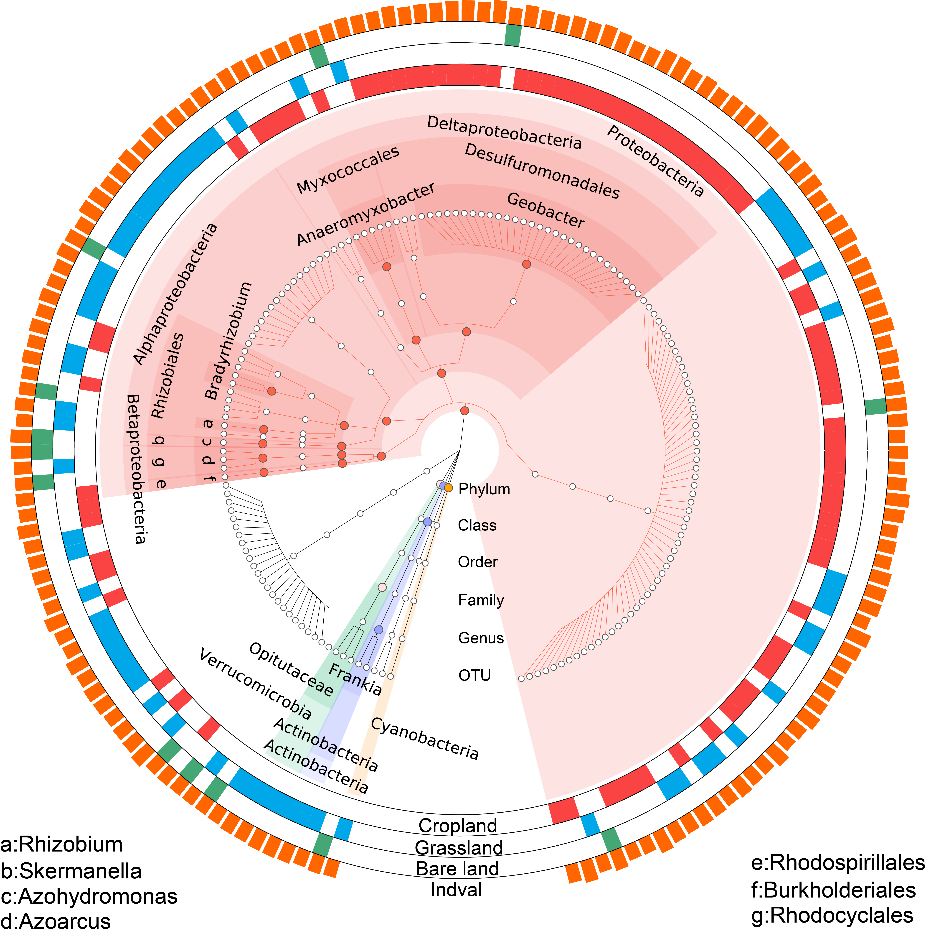


**Supplementary Figure 2.** Indicator OTUs of diazotrophs in different land uses. The outer layer represents Indval values that differ significantly among land uses at *p* < 0.05.

**Supplementary Figure 3.**


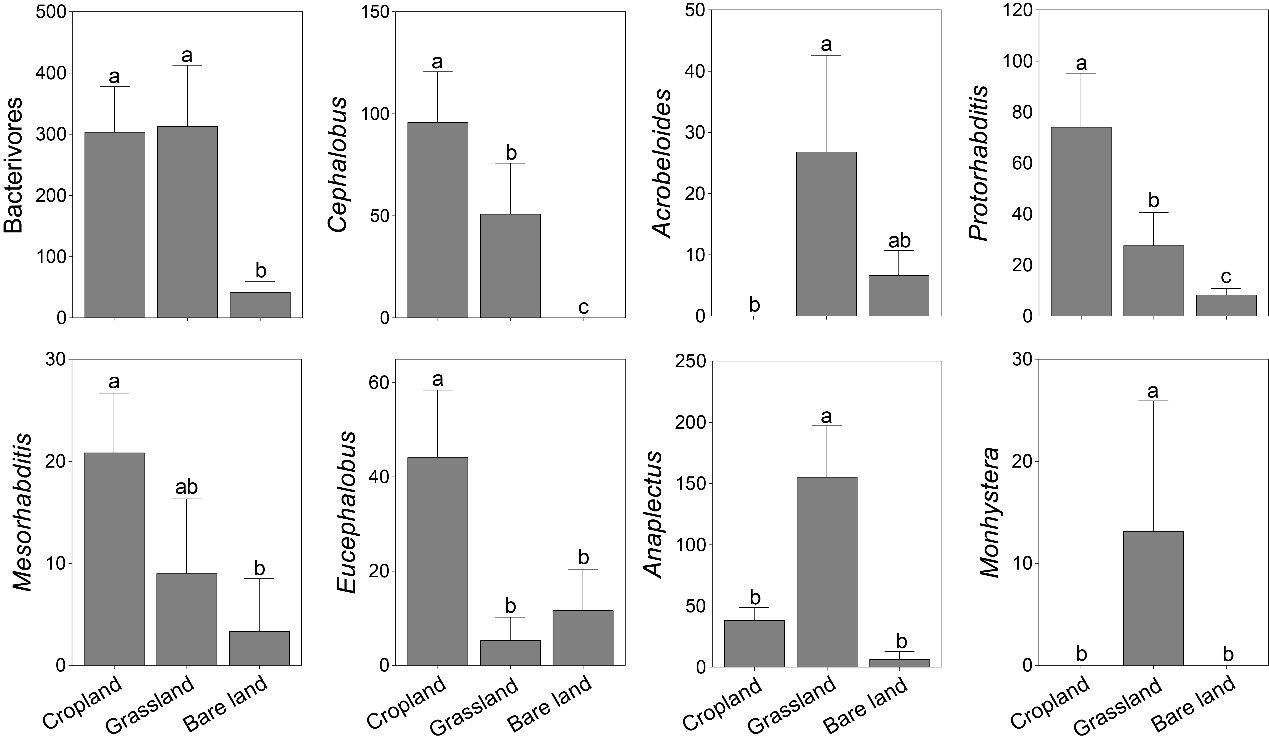


**Supplementary Figure 3.** Abundance of bacterivores and main genera in different land uses. Abundances of bacterivores are presented as individuals per 100 g dry soil. Different lowercase letters indicate significant differences among land uses at *p* < 0.05.

**Supplementary Figure 4.**


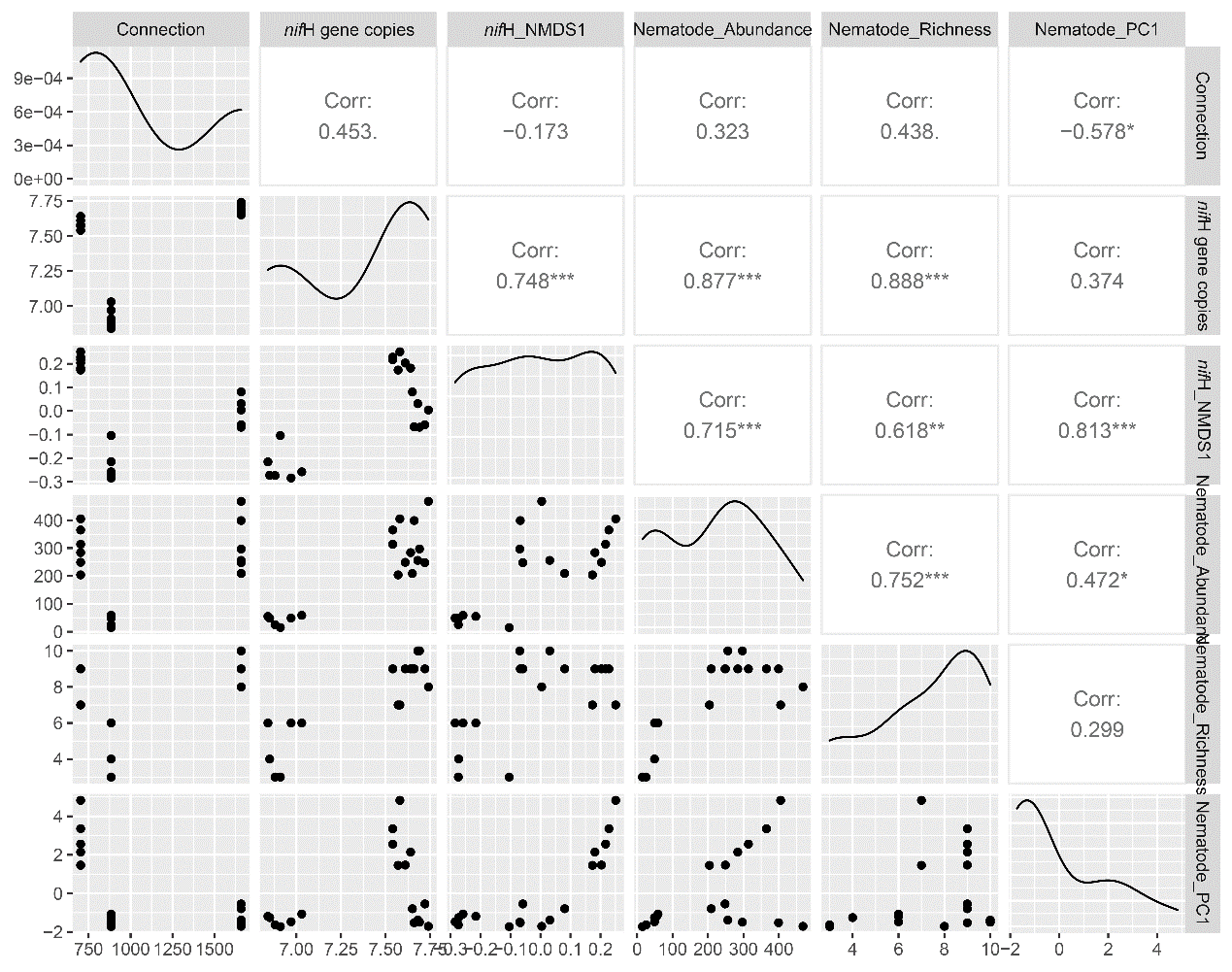


**Supplementary Figure** **4.** Pearson correlation among diazotrophs, bacterivores and parameters of network structure of diazotrophs and bacterivores. Connection, the connection of diazotrophs and bacterivores; *nifH*_NMDS1, the axis 1 of *nifH* gene NMDS; Nematode_Abundance, the abundance of bacterivores; Nematode_Richness; the number of different bacterivore genera, and Nematode_PC1, the PC1 of bacterivore PCA. *, **, and *** represent significant correlation at *p* < 0.05, *p* < 0.01, and *p* < 0.001, respectively.

**Supplementary Figure 5.**

**Supplementary Figure 5.** Soil properties under different land uses. TN, total soil nitrogen; SOC, soil organic carbon; AN, Alkali-hydrolyzable nitrogen. Different lowercase letters indicate significant differences among land uses at *p* < 0.05.
